# An exploratory examination of marijuana use, problem-gambling severity, and health correlates among adolescents

**DOI:** 10.1556/JBA.3.2014.009

**Published:** 2014-04-05

**Authors:** CHRISTOPHER J. HAMMOND, COREY E. PILVER, LOREEN RUGLE, MARVIN A. STEINBERG, LINDA C. MAYES, ROBERT T. MALISON, SUCHITRA KRISHNAN-SARIN, RANI A. HOFF, MARC N. POTENZA

**Affiliations:** ^1^Child Study Center, Yale University School of Medicine, New Haven, CT, USA; ^2^Department of Psychiatry, Yale University School of Medicine, Connecticut Mental Health Center, New Haven, CT, USA; ^3^Department of Biostatistics, Yale University School of Public Health, New Haven, CT, USA; ^4^Problem Gambling Services, Middletown, CT, USA; ^5^Connecticut Council on Problem Gambling, Clinton, CT, USA; ^6^Department of Epidemiology, Yale University School of Medicine, New Haven, CT, USA; ^7^Department of Neurobiology, Yale University School of Medicine, New Haven, CT, USA

**Keywords:** marijuana, gambling, at-risk/problem gambling, adolescence, risk behaviors

## Abstract

*Background and aims:* Gambling is common in adolescents and at-risk and problem/pathological gambling (ARPG) is associated with adverse measures of health and functioning in this population. Although ARPG commonly co-occurs with marijuana use, little is known how marijuana use influences the relationship between problem-gambling severity and health- and gambling-related measures. *Methods:* Survey data from 2,252 Connecticut high school students were analyzed using chi-square and logistic regression analyses. *Results:* ARPG was found more frequently in adolescents with lifetime marijuana use than in adolescents denying marijuana use. Marijuana use was associated with more severe and a higher frequency of gambling-related behaviors and different motivations for gambling. Multiple health/functioning impairments were differentially associated with problem-gambling severity amongst adolescents with and without marijuana use. Significant marijuana-use-by-problem-gambling-severity-group interactions were observed for low-average grades (OR = 0.39, 95% CI = [0.20, 0.77]), cigarette smoking (OR = 0.38, 95% CI = [0.17, 0.83]), current alcohol use (OR = 0.36, 95% CI = [0.14, 0.91]), and gambling with friends (OR = 0.47, 95% CI = [0.28, 0.77]). In all cases, weaker associations between problem-gambling severity and health/functioning correlates were observed in the marijuana-use group as compared to the marijuana-non-use group. *Conclusions:* Some academic, substance use, and social factors related to problem-gambling severity may be partially accounted for by a relationship with marijuana use. Identifying specific factors that underlie the relationships between specific attitudes and behaviors with gambling problems and marijuana use may help improve intervention strategies.

## INTRODUCTION

Gambling is a common recreational activity, especially among adolescents where approximately 77–83% of American youth gamble (Shaffer, Forman, Scanlan & Smith, 2000). While most people gamble without problems, some develop functional impairment related to their gambling. Pathological gambling (PG) is characterized by persistent and recurrent maladaptive patterns of gambling associated with significant social functional impairments, legal difficulties, and psychiatric distress. Similar to other addictive disorders, PG is typified by a compulsive behavior (i.e. compulsive gambling) and is associated with poor self-control over gambling and distortions in thinking about gambling (Chambers & Potenza, 2003). Some individuals experience less severe patterns of gambling (defined as at-risk and problem gambling) that are nonetheless associated with functional impairment, albeit generally not to the extent of PG (Desai, Desai & Potenza, 2007; Desai, Maciejewski, Pantalon & Potenza, 2005). When grouped together, at-risk, problem, and pathological gambling (described collectively as at-risk/problem gambling [ARPG]) is two- to four-fold higher in adolescence and young adulthood than in later adulthood, with a meta-analysis finding a prevalence of 21% in adolescents (Potenza et al., 2011; Rahman et al., 2012; Shaffer, Hall & vander Bilt, 1999). In adolescents and adults, ARPG and PG are associated with poor socio-rela-tional and vocational functioning and co-occurring psychiatric disorders (especially substance-use disorders [SUDs]) (Argo & Black, 2004; Desai & Potenza, 2008; Ellenbogen, Gupta & Derevensky, 2007; Jackson, Dowling, Tomas, Bond & Patton, 2008; Petry, Stinson & Grant, 2005; Shaffer & Korn, 2002; Yip, White, Grilo & Potenza, 2011).

Marijuana use is common in adolescence. Among high school seniors, 43% report lifetime marijuana use with 6% reporting near-daily use (Johnston, O’Malley, Bachman & Schulenberg, 2011). Adolescent marijuana use is associated with neurobiological, psychosocial, and health-related measures including elevated levels of mood and anxiety disorders, externalizing behaviors, suicidality, and risk-taking behaviors (Dorard, Berthoz, Phan & Corcos, 2008; Medina, Nagel, Park, McQueeny & Tapert, 2007; Monshouwer et al., 2007; Pedersen, 2008; Schepis et al., 2011). Marijuana use during adolescence has been linked to neurocognitive deficits and development of mood, anxiety, psychotic, and non-marijuana SUDs (Di Forti, Morrison, Butt & Murray, 2007; Fergusson, Boden & Horwood, 2008; Georgiades & Boyle, 2007; Patton et al., 2007; Wittchen et al., 2007). Adolescent marijuana use is associated with deviant peer group affiliation, poorer grades, and increased rates of school dropout (Bray, Zarkin, Ringwalt & Qi, 2000; Brook, Stimmel, Zhang & Brook, 2008; Leatherdale, Hammond & Ahmed, 2008; Reboussin, Hubbard & Ialongo, 2007).

Gambling behaviors and ARPG commonly co-occur with SUDs in adolescence and adulthood, suggesting shared etiologies (Chambers & Potenza, 2003). In adults with SUDs, the lifetime prevalence of gambling disorders is 29% (compared to 5% in the general population) (Shaffer et al., 1999; Shaffer & Hall, 2001), and substance abusers with co-occurring gambling disorders have greater severity of substance-use problems and increased depression, anxiety, somatization, and social impairment (Ciarrocchi, 1987; Feigelman, Kleinman, Lesieur, Millman & Lesser, 1995; McCormick, 1993; Petry, 2000; Spunt, Dupont, Lesieur, Liberty & Hunt, 1998; Steinberg, Kosten & Rounsaville, 1992). Taken together, ARPG and marijuana use each associate with negative measures in adolescents, and there is a need for better understanding their relationships and potentially interactive influences on health and functioning.

Drug use may interact with gambling behaviors, impacting the severity of both gambling behaviors and substance use and effecting clinical care and treatment outcomes (Grant, Potenza, Weinstein & Gorelick, 2010; Leeman & Potenza, 2012). To date, few studies have focused on the relationship between marijuana use and ARPG, despite multiple studies suggesting marijuana use is common among individuals with gambling problems (Barnes, Welte, Hoffman & Tidwell, 2009; Goldstein, Walton, Cunningham, Resko & Duan, 2009; Huang, Jacobs, Derevensky, Gupta & Paskus, 2007; Kausch, 2003; Proimos, DuRant, Pierce & Goodman, 1998; Westphal, Rush, Stevens & Johnson, 2000). Amongst treatment-seeking adolescents with cannabis-use disorders, ARPG was common (22%) and associated with drug use, legal difficulties, psychiatric problems, and risk-taking behaviors (Petry & Tawfik, 2001). To our knowledge, no previous study has systematically examined the interactions of gambling, marijuana use, and social/academic functioning.

In the current study, we examined relationships between problem-gambling severity, health/social-functioning, and gambling measures in groups distinguished by self-reported marijuana use. We analyzed high school survey data that have been used previously to investigate correlates of problem-gambling severity in general and in relation to age of gambling onset, lottery ticket gift receipt and Internet gambling (Kundu et al., 2013; Potenza et al., 2011; Rahman et al., 2012; Yip et al., 2011). Here we adopt a similar strategy to examine health, functioning, and gambling measures with respect to problem-gambling severity and marijuana use. We hypothesized that marijuana use as compared to non-use would more likely associate with ARPG, different gambling patterns, different gambling-related motivations, heavy gambling, and an earlier age at gambling onset. Based on findings in adults with nicotine- and alcohol-use disorders in which associations between ARPG and psychopathology appeared partially accounted for by nicotine dependence and alcohol abuse/dependence (Brewer, Potenza & Desai, 2010; Grant, Desai & Potenza, 2009), we hypothesized that ARPG would be less strongly associated with substance use, aggression, dysphoria/depression, and poor academic performance in the marijuana-use group as compared to marijuana-non-use group.

## METHODS

### Study design and sampling

Survey design, recruitment, and methodology have been described previously (Cavallo et al., 2010; Desai, Krishnan-Sarin, Cavallo & Potenza, 2010; Grant et al., 2010; Grant, Potenza, Krishnan-Sarin, Cavallo & Desai, 2011; Liu, Desai, Krishnan-Sarin, Cavallo & Potenza, 2011; Potenza et al., 2011; Rahman et al., 2012; Schepis et al., 2008, 2011; Yip et al., 2011). Briefly, all public four-year and non-vocational or special education high schools in Connecticut were invited to participate. Schools were offered a report that outlined the prevalence of the queried behaviors in that school. Schools that expressed an interest in participating were contacted and permission was obtained from School boards. Secondary targeted selection was conducted to ensure that the final sample was geographically representative and contained schools from each of the three tiers of the state’s district reference groups (DRGs). DRGs are groupings of schools based on the socioeconomic status of families in the school district. Although not a random sample of public high school students, the sample is similar in demographics to the Connecticut residents aged 14–18 from the 2000 Census.

Surveys were administered by research staff in school-wide assemblies or English/Health classes. Students were informed that participation was voluntary, were given pens for participating, and were instructed not to write identifying information on the survey. Refusal rate for participation was less than 1%. Ineligible students worked quietly on other schoolwork while other students completed the survey.

### Demographic and health/functioning variables

Sociodemographic variables including gender, age, ethnicity/race, grade level, and family structure were assessed, as were high school grade average and engagement in extracurricular activities. Questions assessing function were adopted from widely used surveys (e.g., Youth Health Risk Behavior Survey). Past-year dysphoria/depression was defined as having endorsed feeling “so sad or hopeless almost every day for 2 weeks or more in a row that stopped you from doing some usual activities”. Survey items assessing aggressive behaviors queried whether a respondent carried a weapon within the past 30 days and whether a respondent got into a physical fight within the past year, with both questions coded dichotomously as “yes/no”.

### Substance-use variables

Lifetime marijuana use was assessed with the question, “Have you ever smoked marijuana?” and coded dichoto-mously as “yes/no”. Respondents were categorized as lifetime marijuana users if they responded “yes” to that question and as lifetime marijuana non-users if they responded “no”. Marijuana use behaviors were also assessed by querying past 30-day marijuana use, frequency of marijuana use, age at first use of marijuana, and the social environment in which marijuana was smoked (i.e. with friends, family, strangers, or alone). Lifetime cigarette smoking was coded into three categories: never, occasionally, or regularly. Lifetime alcohol and lifetime other drug use were coded dichotomously “yes/no”. Current alcohol use was coded into one of four categories: not regular (1–5 days in the past 30 days), light (6–9 days), moderate (10–19 days), and heavy (20–30 days). Caffeine use was classified into one of three categories: none, 1–2 caffeinated beverages per day, and 3 or more caffeinated beverages per day.

### Gambling variables

Questions assessing gambling behaviors, characteristics, and substance use were adapted from those used in prior gambling surveys and included questions from the Massachusetts Gambling Screen (MAGS) (Shaffer, LaBrie, Scanlan & Cummings, 1994) and the Gambling Impact and Behavior Study (Gerstein, Hoffmann & Larison, 2002). The MAGS is a 12-item validated clinical screening instrument generated to assess DSM-IV PG criteria in adolescents with high reliability (alpha = 0.87) and strong discriminant predictive validity (Shaffer et al., 1994). Gambling measurements queried gambling types, locations, frequency, gambling partners, and motivations. Types of gambling were assessed by asking about lottery/scratch card, dice/craps, machine gambling, and placing bets with a bookie. Internet gambling was defined as having placed a bet on the Internet. Machine gambling was defined as any gambling on a poker machine or slot machine, and placing bets on a video or arcade game, but did not include Internet gambling. Gambling motivations were assessed with responses grouped into four categories: for fun/excitement, escape or to relieve dyspho-ria, financial reasons, and social reasons. Additional items queried respondents about locations where they gambled and whether they experienced gambling-related anxiety or pressure. Respondents were queried regarding gambling with family, friends, other adults, and strangers, and whether a respondent gambled alone. Age at first gamble and average time spent gambling per week were also assessed.

### Gambling groups

Gambling was defined as “any game you bet on for money OR anything else of value”. Respondents were stratified into groups based upon past-year gambling status. Among the respondents who endorsed past-year gambling, problem gambling severity groups were determined based upon DSM-IV criteria (e.g. preoccupation with gambling, unsuccessful effort to control gambling, needing to gambling with increasing amounts of money in order to achieve desired excitement, jeopardized or lost a significant relationship, job, or career opportunity because of gambling) as assessed using items from the MAGS. Only data from subjects providing information on both marijuana use and DSM-IV criteria items from the MAGS used to determine problem-gambling severity were used in analyses. Among the 4,523 subjects surveyed [2124 males (47.5%) and 2345 (52.5%) females], 2,252 (49.8%) were included in analyses. Of the adolescents with missing data, 1839 (81.0%) were missing information on ARPG designation, 232 (10.2%) were missing information on marijuana use, and 200 (8.8%) were missing information on both ARPG and marijuana use. Completers included 1232 males (55.5%) and 987 (44.5%) females. Baseline demographic data were evaluated for differences between those with complete data and those without complete data using Chi-square analyses. Students with complete data were more likely to be male (*χ*^2^ = 112.91, *p* < 0.001), older (*χ*^2^ = 16.38, *p* < 0.001), in a later grade (i.e., 11^th^ or 12^th^ grade) (*χ*^2^ = 19.23, *p* < 0.001), have a lower grade point average (i.e., more likely to have C’s or D’s and F’s) (*χ*^2^ = 21.03, *p* < 0.001), and of Caucasian race (*χ*^2^ = 19.88, *p* < 0.001) and were less likely to be of Asian (*χ*^2^ = 9.84, *p* = 0.002) or ‘Other’ (*χ*^2^ = 6.75, *p* = 0.009) race. Study subjects who reported past-year gambling but did not acknowledge DSM-IV criteria for PG were classified as having low-risk gambling (LRG). Subjects endorsing one or more DSM-IV criteria were classified as having at-risk/problem gambling (ARPG). Subjects who did not endorse past-year gambling were categorized as being non-gambling (NG). These severity groups have been used in prior studies of adolescent and adult gamblers (Brewer et al., 2010; Desai & Potenza, 2008; Potenza et al., 2011; Rahman et al., 2012).

### Data analysis

Statistical analyses were conducted using the SAS system 2.0 (SAS Institute, Cary, NC, USA). Using bivariate analyses, we first examined associations between problem-gambling severity and socio-demographic variables stratified by lifetime marijuana use. Next, we examined the relationships among lifetime marijuana use, problem-gambling severity level, and gambling-related behavioral measures using chi-square tests. Finally, we constructed stratified binomial and multinomial logistic regression models to generate the marijuana-use-specific effects of problem-gambling severity, adjusted for relevant socio-demographic covariates (gender, race, Hispanic ethnicity, grade level, and family structure). In the logistic regression models, health/functioning and gambling measures were the dependent variables of interest, and problem-gambling severity, marijuana use, and the marijuana-use-by-problem-gambling-severity interaction were the independent variables of interest. Outcome data presented includes marijuana-use-specific odds ratios (ORs) and marijuana-use-by-problem-gambling-severity interaction odds ratios (IORs) and their associated 95% confidence intervals (95% CIs). Statistical significance was considered to be *p* < .05. As this study was exploratory, an arguably overly conservative statistical significance thresholding procedure (e.g., Bonferroni correction) was not employed.

### Ethics

For permission, a passive consent procedure was utilized. Letters were sent to parents providing information about the study and informing them to contact the school directly if they wished to deny permission for their child/children to participate. Students were given the option of not participating. The passive consent procedure was approved by participating schools. This study was approved by the Yale University School of Medicine Institutional Review Board.

## RESULTS

### Sociodemographic characteristics

Sociodemographic characteristics of the sample are presented (Table 1). Among marijuana-using adolescents, problem-gambling severity was significantly associated with gender, race, and family structure (*p* < .05). Among marijuana-non-using adolescents, problem-gambling severity was associated with gender, race, and grade level (*p* < 0.05).

**Table 1.** Socio-demographic characteristics, by marijuana-use status and problem-gambling severity

Variable/Category	Lifetime marijuana use (%)			χ^2^ Statistics		Lifetime marijuana non-use (%)			χ^2^ Statistics	
	Non-gambling (*N* = 116)	Low-risk gambling (*N* = 529)	At-risk/problem gambling (*N* = 348)	χ^2^	*p*	Non-gambling (*N* = 276)	Low-risk gambling (*N* = 704)	At-risk/problem gambling (*N* = 279)	χ^2^	*p*
	%	%	%			%	%	%		
Gender				115.11	<0.001				85.51	<0.001
Male	32.2	48.8	79.6			34.9	53.3	74.4		
Female	67.8	51.2	20.4			65.1	53.3	25.6		
Race/Ethnicity										
Caucasian	75.0	80.2	68.1	16.40	<0.001	60.5	71.7	68.1	11.60	0.003
African American	7.8	8.7	17.5	17.83	<0.001	15.6	8.4	12.2	11.38	0.003
Asian	1.7	3.0	6.0	6.73	0.04	9.1	5.0	5.0	6.46	0.04
Hispanic	19.1	12.7	20.1	9.01	0.01	14.2	16.3	19.2	2.37	0.31
Other	16.4	12.9	14.7	1.25	0.54	18.5	18.2	21.2	1.19	0.55
Grade				9.48	0.15				15.20	0.02
9^th^	19.1	20.5	26.5			27.9	34.7	37.8		
10^th^	21.7	24.1	25.9			24.6	28.8	27.3		
11^th^	38.3	32.6	26.8			31.9	23.0	20.1		
12^th^	20.9	22.8	20.8			15.6	13.6	14.8		
Family structure				12.81	0.01				6.47	0.17
One parent	32.5	29.2	24.8			16.7	19.5	18.3		
Two parents	58.8	65.2	63.4			77.0	75.1	72.9		
Other	8.8	5.6	11.8			6.3	4.9	8.8		

### Problem-gambling severity and marijuana use

Lifetime marijuana use was reported by 993 (44.1%) respondents. Of adolescents reporting lifetime marijuana use, 614 (63.2%) reported use of marijuana in the 30 days prior to taking the survey; additionally, 313 (32.7%) reported no regular use, 253 (26.1%) reported using once or twice per week, 141 (14.2%) reported using 3–6 times per week, and 250 (25.2%) reported daily use.

Of the 2252 respondents, 392 (17.4%) were NG, 1233 (54.8%) had LRG, and 627 (27.8%) had ARPG. Problem-gambling severity was associated with marijuana use (*χ*^2^= 67.33, *p* < 0.001); the percentage with ARPG was greater in the marijuana-use group compared to the non-use group (35.0% vs. 22.1%).

### Gambling behaviors and motivations

In bivariate analysis, marijuana use versus non-use was associated with non-strategic and machine gambling and gambling online, at school, and in a casino (Table 2). Marijuana use versus non-use was associated with feelings of an urge or pressure to gamble, gambling-related tension/anxiety before gambling that was subsequently relieved by gambling, gambling to escape or relieve dysphoria, gambling for financial reasons, and gambling for social reasons. Marijuana use versus non-use was associated with gambling with friends, strangers, and alone, spending more hours per week gambling, and earlier age of gambling onset.

**Table 2.** Gambling measures by marijuana-use status

Variable/Category	Lifetime marijuana use status		*χ*^2^ statistics	
	Use (*N* = 993)	Non-use (*N=* 1259)	*χ*^2^	*p*
	%	%		
Gambling type				
Strategic	96.1	95.5	0.41	0.52
Non-strategic	74.5	67.3	11.32	<0.001
Machine	55.8	40.3	44.49	<0.001
Gambling location				
Online	25.4	15.0	31.11	<0.001
School gambling	51.8	29.2	98.40	<0.001
Casino	16.8	4.8	70.65	<0.001
Gambling triggers				
Pressure/Urge	10.9	6.9	9.41	0.002
Anxiety	9.6	3.2	29.44	<0.001
Gambling motivations				
Excitement	68.0	67.0	0.22	0.64
Financial reasons	59.9	48.3	24.84	<0.001
Escape	34.3	26.0	15.14	<0.001
Social reasons	41.2	35.2	7.00	0.008
Gambling partners				
Family	44.1	45.8	0.51	0.48
Friends	74.5	67.9	9.81	0.002
Other adults	26.0	22.9	2.43	0.12
Strangers	14.5	3.9	64.61	<0.001
Alone	10.5	6.8	8.00	0.005
Time spent gambling			35.96	<0.001
1 hour or less per week	77.2	88.5	
2 or more hours per week	22.9	11.6		
Age of onset of gambling			13.42	0.004
<8 years	18.3	12.0		
9–11 years	16.6	16.6		
12–14 years	33.7	39.7		
15+ years	31.5	31.6		

In multivariate-adjusted logistic regression models, ARPG adolescents were more likely than LRG adolescents to engage in strategic, non-strategic, and machine gambling; to gamble online, at school, and at a casino; to have motivations of excitement, escape/reduction of dysphoria, financial reasons, and social reasons to gamble; to gamble to reduce pressure or anxiety; to gamble with family, other adults, strangers, and alone; and to spend significantly more time gambling (Table 3). These associations were observed in both the marijuana-use and marijuana-non-use groups. In the marijuana-using group, ARPG adolescents were less likely than LRG adolescents to have ages of gambling onset older than 12 years. A significant marijuana-use-by-problem-gambling-severity interaction was observed for gambling with friends. In the marijuana-non-using group, ARPG adolescents were more likely than LRG adolescents to gamble with friends; this association was not observed in the marijuana-using group. No other interactions were observed between marijuana-use status and problem-gambling severity level.

**Table 3.** Gambling measures by marijuana-use status and problem-gambling severity

Variable/Category	Lifetime marijuana use			Lifetime marijuana non-use			Interaction odds ratio marijuana use vs. marijuana non-use
	Low-risk gambling (*N* = 529)	At-risk/ Problem gambling (*N* = 348)	At-risk/Problem gamblers vs. low-risk gambling		Low-risk gambling (*N* = 704)	At-risk/Problem gambling (*N* = 279)	At-risk/Problem gamblers vs. low-risk gambling
	%	%	OR (95% CI)	%	%	OR (95% CI)	OR (95% CI)
Gambling type							
Strategic	94.5	98.6	3.07 (1.12-8.40)*	94.2	98.9	7.60 (1.77-32.62)**	0.52 (0.09-2.96)
Non-strategic	71.8	78.5	1.80 (1.24-2.61)**	66.1	70.6	1.47 (1.05-2.06)*	1.21 (0.75-1.96)
Machine	44.1	73.6	2.53 (1.82-3.52)***	35.1	53.4	1.83 (1.35-2.49)***	1.44 (0.92-2.23)
Gambling location							
Online	14.9	41.4	3.50 (2.44-5.03)***	10.6	25.9	2.38 (1.60-3.55)***	1.46 (0.87-2.46)
School gambling	36.4	75.4	4.30 (3.03-6.11)***	19.9	52.9	3.84 (2.73-5.39)***	1.17 (0.73-1.90)
Casino	8.2	30.1	4.44 (2.79-7.06)***	3.4	8.3	2.80 (1.47-5.33)**	1.88 (0.88-4.02)
Gambling triggers							
Pressure/Urge	4.2	21.4	5.30 (3.02-9.30)***	3.6	15.3	4.20 (2.42-7.30)***	1.10 (0.52-2.34)
Anxiety	1.9	21.2	12.37 (5.27-29.04)***	0.5	9.9	32.75 (7.46-143.73)*** 0.44(0.08-2.33)	
Gambling motivations							
Excitement	58.6	82.2	3.11 (2.16–4.48)***	60.9	82.1	2.67 (1.84-3.88)***	1.10 (0.66-1.82)
Financial reasons	46.9	79.6	3.89 (2.75-5.49)***	40.5	68.1	2.77 (2.01-3.82)***	1.34 (0.85-2.12)
Escape	24.6	49.1	2.91 (2.10–4.04)***	20.03	91.2	2.74 (1.98-3.80)***	1.00 (0.65-1.56)
Social reasons	33.5	52.9	2.05 (1.50-2.80)***	30.3	47.7	1.93 (1.40-2.67)***	1.02 (0.67-1.57)
Gambling partners							
Family	39.3	51.4	1.54 (1.13-2.09)**	42.5	54.1	1.47 (1.09-1.99)*	0.99 (0.65-1.49)
Friends	71.8	78.5	1.18 (0.82-1.71)	62.6	81.0	2.31 (1.59-3.37)***	0.47 (0.28-0.77)**
Other adults	17.0	39.7	2.78 (1.96-3.95)***	19.2	32.7	2.10 (1.48-2.92)***	1.38 (0.86-2.19)
Strangers	5.3	28.5	5.13 (3.10-8.52)***	1.9	9.0	3.69 (1.77-7.69)***	1.31 (0.56-3.10)
Alone	5.7	17.8	2.36 (1.40–4.00)**	3.7	14.7	4.36 (2.48-7.65)***	0.67 (0.32-1.39)
Time spent gambling†							
2 hours or more per week	9.2	41.6	6.22 (3.95-9.80)***	6.6	23.0	3.54 (2.19-5.74)***	1.62 (0.85-3.10)
Age of onset of gambling‡							
9–11 years	13.8	20.1	0.93 (0.53-1.63)	15.8	18.2	1.07 (0.59-1.95)	0.94 (0.42-2.08)
12–14 years	36.2	30.5	0.51 (0.31-0.83)**	38.9	41.3	0.95 (0.56-1.60)	0.58 (0.29-1.15)
15+ years	36.7	25.0	0.55 (0.33-0.92)*	34.0	27.0	0.63 (0.36-1.12)	0.82 (0.39-1.73)

*Note:* We first constructed stratified binomial and multinomial logistic regression models to generate the marijuana-use-specific effects of problem-gambling severity, adjusted for relevant socio-demographic covariates (gender, race, Hispanic ethnicity, grade level and family structure). We then fit logistic regression models using the full analytical sample; models included the main effects of marijuana use and problem-gambling severity, as well as the marijuana-use-by-problem-gambling-severity interaction. Relevant socio-demographic covariates were included in the models as well. Presented are marijuana use-specific odds ratios (ORs) and their associated 95% confidence intervals (95% CIs); the OR quantifies the magnitude and direction of the association between problem-gambling severity and gambling behaviors and motivations. Furthermore, a 95% CI that does not in-clude 1.0 indicates a statistically significant association. Also presented are interaction odds ratios (IORs) and their associated 95% CIs. The IOR is the ratio of the marijuana-use-specific odds ratios (i.e., OR [use]/OR [non-use]); in this case, a 95% CI that does not include 1.0 indicates that marijuana use moderates the association between problem-gambling severity and the outcome of interest. * *p* < 0.05, ** *p* < 0.01, *** *p* < 0.001; ^†^ Referent category = 1 hour or less per week; ^‡^ Referent category = ≤8 years

### Health/functioning measures

The associations between problem-gambling severity and measures of health and functioning are presented in Table 4. In both marijuana-using and marijuana-non-using groups, elevated odds were observed in association with greater levels of problem-gambling severity (for LRG versus NG and/or ARPG versus NG) for measures of cigarette smoking, alcohol use, other drug use, caffeine use, aggression, depression, and participation in extracurricular activities (Table 4). Significant marijuana-use-by-gambling-group interactions were observed for occasional cigarette smoking versus never smoking (at the level of ARPG), light current alcohol use versus never regular use (at the level of ARPG), and C grade average versus A’s and B’s (at the level of LRG). In each case, the magnitude of the associations between problem-gambling severity and the outcome of interest was lower in the marijuana-using group as compared to the non-using group. No other marijuana-use-by-problem-gambling-severity interactions were observed, indicating the associations between problem-gambling severity and these measures of health and well-being are similar in the marijuana-using and marijuana-non-using groups.

**Table 4.** Health and functioning measures by marijuana-use status and problem-gambling severity

Variable/Category	Lifetime marijuana use **(%)**			Adjusted odds ratios marijuana use		Lifetime marijuana non-use (%)			Adjusted odds ratios marijuana non-use		Interaction odds ratios marijuana use vs. non-use	
	Non-gambling (*N* = 116)	Low-risk gambling (*N* = 529)	At-risk/ Problem gambling (*N* = 348)	Low-risk gambling vs. non-gambling	At-risk/ Problem gambling vs. non-gambling	Non-gambling (*N* = 276)	Low-risk gambling (*N* = 704)	At-risk/ Problem gambling (*N* = 279)	Low-risk gambling vs. non-gambling	At-risk/ Problem gambling vs. non-gambling	Low-risk gambling vs. non-gambling	At-risk/ Problem gambling vs. non-gambling
	**%**	**%**	**%**	OR (95% CI)	OR (95% CI)	**%**	**%**	**%**	OR (95% CI)	OR (95% CI)	OR	OR
**Substance use**												
*Cigarette smoke*†												
Occasionally	41.4	43.9	38.6	1.30 (0.75-2.25)	1.53 (0.83-2.82)	8.2	14.7	21.4	2.17 (1.31-3.61)**	4.07 (2.31-7.16)***	0.61 (0.29-1.27)	0.38 (0.17-0.83)*
Regularly	33.3	34.3	35.0	1.41 (0.78-2.56)	2.36 (1.23-.55)**	1.5	2.6	4.4	2.12 (0.69-6.54)	3.58 (1.04-12.35)*	0.74 (0.21-2.59)	0.62 (0.16-2.37)
Alcohol use. Lifetime	91.2	98.0	96.2	5.52 (2.17-14.08)***	4.14 (1.52-11.23)**	62.7	84.2	79.8	3.91 (2.68-5.72)*	3.68 (2.29-5.91)*	1.59 (0.59-4.27)	1.48 (0.54-1.07)
*Current alcohol frequency*‡												
Light	33.7	23.8	23.8	0.69 (0.34-1.39)	0.89 (0.40-1.97)	26.5	32.6	32.2	1.89 (0.99-3.60)	2.05 (0.98^1.32)	0.36 (0.14-0.91)*	0.40 (0.14-1.10)
Moderate	34.8	39.5	36.2	1.22 (0.61-2.45)	1.73 (0.79-3.78)	10.3	20.5	23.7	2.64 (1.11-6.27)*	3.33 (1.29-8.12)*	0.40 (0.14-1.20)	0.35 (0.11-1.14)
Heavy	12.0	18.8	24.2	2.42 (0.95-6.18)	5. (1.86-14.20)**	1.5	5.2	6.6	5.15 (0.66-40.41)	8.56 (1.00-72.91)*	0.47 (0.05-4.43)	0.63 (0.06-6.53)
Other drug use. Lifetime	22.5	25.8	38.4	1. (0.73-2.45)	2.27 (1.20-4.29)*	0.4	0.7	4.6	1.99 (0.21-18.83)	13.32 (1.49-119.23)*	0.86 (0.09-8.36)	0.03 (0.03-2.39)
*Caffeine use*§												
1-2 per day	46.2	49.9	39.7	2.06 (1.17-3.62)**	1.68 (0.89-3.14)	54.8	55.5	45.5	1.40 (0.97-2.01)	1.08 (0.68-1.70)	1.42 (0.74-2.73)	1.34 (0.65-2.77)
3+ per day	24.5	34.6	42.7	2.58 (1.38-4.82)**	2.99 (1.51-5.91)***	12.4	23.8	31.7	2.69 (1.63-1.46)***	3.25 (1.81-5.82)***	0.93 (0.42-2.05)	0.82 (0.35-1.92)
**Aggression**												
Serious fights	8.3	11.6	27.1	1.55 (0.69-3.47)	3.30 (1.45-7.48)**	1.9	3.3	10.6	2.12 (0.71-6.35)	5.76 (1.87-17.72)**	0.79 (0.21-3.03)	0.65 (0.17-2.49)
Carry weapon	23.6	30.4	57.6	1.57 (0.90-2.73)	3.46 (1.94-6.17)***	4.9	16.5	28.6	2.93 (1.55-5.56)***	4.83 ^;^ (2.47-9.46)***	0.49 (0.21-1.12)	0.62 (0.26-1.48)
**Mood**												
Dysphoria/	28.6	28.3	33.2	1.09	1.74	16.2	16.4	23.7	1.34	2.86	0.91	0.81
Depression				(0.66-1.79)	(1.00-3.01)*				(0.86-2.06)	(1.70-4.80)***	(0.47-1.76)	(0.40-1.64)
**Academic/ Extracurricular**												
Grade average												
A's and B's	37.7	42.0	36.9	–	–	75.7	65.1	56.6	–	–	–	–
Mostly C's	39.5	39.3	37.2	0.83 (0.51-1.36)	0.77 (0.45-1.32)	18.4	27.3	31.5	1.74 (1.18-2.58)**	2.06 (1.31-3.26)**	0.52 (0.28-0.96)*	0.39 (0.20-0.77)**
D's or lower	22.8	18.8	25.9	0.77 (0.42-1.42)	0.89 (0.46-1.72)	6.0	7.6	12.0	1.23 (0.66-2.32)	1.97 (0.98-3.98)	0.67 (0.28-1.60)	0.49 (0.20-1.23)
Extracurricular activities	57.8	65.5	73.9	1.23 (0.79-1.91)	1.64 (1.00-2.69)	73.9	83.8	82.4	1.89 (1.34-2.72)***	2.04 (1.28-3.25)**	0.69 (0.40-1.21)	0.98 (0.51-1.87)

*Note:* We first constructed stratified binomial and multinomial logistic regression models to generate the marijuana-use-specific effects of problem gambling severity, adjusted for relevant socio-demographic covariates (gender, race, Hispanic ethnicity, grade level, and family structure). We then fit logistic regression models using the full analytical sample; models included the main effects of marijuana use and problem gambling severity, as well as the marijuana use-by-problem-gambling severity interaction. Relevant socio-demographic covariates were included in the models as well. Presented are marijuana use-specific odds ratios (ORs) and their associated 95% confidence intervals (95% CIs); the OR quantifies the magnitude and direction of the association between problem gambling severity and health and functioning measures. Furthermore, a 95% CI that does not include 1.0 indicates a statistically significant association. Also presented are interaction odds ratios (IORs) and their associated 95% CIs. The IOR is the ratio of the marijuana-use specific odds ratios [i.e., OR (users)/OR (nonusers)]; in this case, a 95% CI that does not include 1.0 indicates that marijuana use moderates the association between problem gambling severity and the outcome of interest. *p < 0.05, ** *p* < 0.01, *** *p* < 0.001; ^†^ Referent category = Never; ^‡^ Referent category = Never regular;^§^ Referent category = None.

## DISCUSSION

### Summary

To our knowledge, this is the first study to systematically examine relationships between adolescent marijuana use, problem-gambling severity and a range of health/functioning and gambling-related measures in a large sample of adolescents. Marijuana-using and non-using adolescents differed on types and locations of gambling, motivations for gambling, time spent gambling and age at gambling onset. Marijuana-use status moderated the relationships between problem-gambling severity and several gambling-related behaviors and health/functioning measures. Specifically, marijuana use moderated the relationships between problem-gambling severity and light-moderate substance use, academic performance and gambling with friends. Health and functional impairments were associated with problem-gambling severity, with marijuana use appearing to account for some of the variance in the associations between ARPG and negative measures of health and functioning.

### Lifetime marijuana use and problem-gambling severity

Consistent with our first hypothesis, adolescents who reported lifetime marijuana use were more likely to report greater problem-gambling severity. The percentage of ARPG in marijuana-using adolescents (35%) is similar to that in adults with SUDs (29%), with estimates appearing to exceed those reported by Petry and Tawfik (2001) who found that 21% of treatment-seeking adolescents with marijuana abuse or dependence met criteria for ARPG. These studies differed with regard to samples (community versus clinical), assessment methodology and related bias (i.e. attrition rates/non-response bias), inclusionary/exclusionary criteria, and determination of problem-gambling status; thus, methodological differences may have contributed to differences in frequencies. Previous studies of adolescents have found associations between gambling, problem-gambling severity, and marijuana use and abuse (Barnes et al., 2009; Goldstein et al., 2009; Kausch, 2003; Petry & Tawfik, 2001; Proimos et al., 1998; Westphal et al., 2000). Barnes et al. (2009) found that rates of heavy gambling were twice as high (36%) for youth who smoked marijuana more than 52 times within the past year as compared to youth who did not smoke marijuana (15%). Future studies should focus on how marijuana use modifies gambling behaviors, specifically in relation to clinical outcomes.

### Gambling behaviors/motivations and health/functioning measures

Consistent with our hypotheses related to gambling behaviors and motivations, marijuana-using and non-using adolescents differed in frequency and severity of gambling behaviors, urges, and motivations to gamble. Marijuana-using adolescents endorsed increased engagement in different gambling types (non-strategic and machine gambling) and across multiple locations (online, school gambling, and casino gambling) compared to their non-using counterparts. Of particular note, over 50% of adolescents reporting marijuana use reported gambling on school grounds compared to close to 30% of marijuana-non-using adolescents. While further studies are needed, these findings suggest that the school setting may be an important location for targeted screening and intervention in adolescents for whom gambling and marijuana use are problematic. Within this framework, schools should consider querying about or assessing for gambling behaviors amongst youth with suspected or identified marijuana-use problems and policies for identifying and preventing gambling on school grounds.

The broader endorsement of gambling participation and motivations in association with marijuana use has multiple clinical implications and may reflect differences in accessibility, acceptance, or willingness to engage in illegal or risky behaviors. Alternatively, as marijuana use was associated with financial motivations to gamble, the possibility exists that gambling winnings might be used to purchase marijuana, although other possibilities exist. The association between marijuana use and gambling to escape raises the possibility that difficulties coping may similarly drive marijuana use and gambling in certain adolescents, although this too warrants additional investigation. Marijuana use was associated with pressure/urges to gamble and subjective anxiety related to gambling, raising the question as to whether marijuana use might be used to target states like gambling-related anxiety or urges. Alternatively, youth prone to anxiety might use both gambling or marijuana to target their anxiety, or gambling or marijuana use may lead to anxieties that perpetuate additional engagement. These relationships warrant additional study. Nonetheless, the findings suggest that screening for gambling-related emotions, motivations and behaviors may be particularly important amongst marijuana-using adolescents.

A marijuana-use-by-problem-gambling severity interaction was noted for gambling with friends, indicating a weaker association amongst marijuana-using adolescents. This finding suggests that social aspects of gambling, namely gambling with friends and in social environments in comparison to gambling alone, may differ in relation to adolescent ARPG with and without marijuana use. Social modeling may influence gambling behavior, especially in the early stages of gambling when behaviors are not problematic. Gupta and Derevensky (1997) found that 75% of pre-adolescents who gambled within the past year gambled with friends and 86% gambled with family members. Initiation of gambling behaviors may occur in social settings with peers and family members and, in some vulnerable adolescents, progress to problematic gambling behaviors. Langhinrichsen-Rohling, Rohde, Seeley and Rohling (2004) performed a functional analysis of gambling behaviors and family and peer correlates in 1846 high school students and found two functional subgroups with ARPG, one of whom reported elevated peer-influence/pressure, peer-gambling behaviors and another who reported limited peer-influence and peer-gambling behaviors.

Multiple hypotheses may explain differences in gambling with friends as related to problem-gambling severity in marijuana-using and non-using groups. Non-marijuana-using adolescents with ARPG may be more prone to gambling-related peer influences and pressure leading to increased social gambling. Gambling social circles and marijuana-using social circles may differ. Marijuana-using adolescents may affiliate primarily with marijuana-using peers and socialize in marijuana-using settings rather than gambling settings, whereas marijuana-non-using adolescents may be more likely to frequent and associate with peers in gambling environments. The socio-environmental contributions to peer interaction and co-occurring gambling, marijuana use and other risk behaviors in adolescents warrant further examination.

Partially consistent with our hypotheses related to health/functioning measures, ARPG was differentially associated with substance use and academic performance in marijuana-using and non-using groups. Marijuana-use-by-problem-gambling-severity interactions were found for occasional cigarette smoking, light current alcohol use, and “C grade” average. Interaction analyses of problem-gambling severity and other measures of health/functioning including aggression, depression, poor academic functioning, and heavy non-cannabis substance use were not moderated by marijuana-use status. The finding that the marijuana-using group more frequently acknowledged impairment across most health and functional measures, and yet the health and functional impairments generally had a weaker association with problem-gambling severity in the marijuana-using group, suggests that some problem-gambling-related health/functioning impairments may be attributable to marijuana use. In other words, marijuana use may account for some of the elevated health/functional impairments associated with ARPG in adolescents, specifically in relation to mild-to-moderate substance-use behaviors and low-average grades. Our results are consistent with previous studies in which adolescent marijuana use has been linked to functional impairments including increased alcohol and tobacco use, lower grade-point average, and high school dropout (Bray et al., 2000; Brook et al., 2008; Leatherdale et al., 2008; Reboussin et al., 2007) and the notion that marijuana use may link to experimentation with a wide range of substances (i.e., as a “gateway” drug). The current findings suggest that some of these “gateway” behaviors may also link to problematic gambling.

### Clinical implications

The data have important implications for public policy, prevention, screening, and clinical care. Screening for and treatment of adolescents with ARPG and comorbid substance use remains limited and fragmented. Adolescents with substance-use problems and comorbid psychiatric disorders (including ARPG) have lower treatment-seeking rates than their adult counterparts, and adolescent mental-health and substance-use service agencies frequently operate in separate systems of care, impeding an integrated approach (Harris & Edlund, 2005; Hawkins, 2009). Public policy should be research-informed and limit availability of gambling and access to gambling venues by adolescents (Messerlian, Derevensky & Gupta, 2005). Increased screening for both adolescent gambling problems and SUDs should occur across multiple settings, as adolescents with functional impairment secondary to gambling and substance use may be identified using brief screening instruments. School- and clinic-based prevention programs should be integrated to target gambling behaviors, substance use, and other risk behaviors concurrently, and prevention and treatment approaches should be developmentally informed. Messerlian et al. (2005) developed the Youth Gambling Risk Prevention Model providing a framework for prevention, intervention, and treatment across different levels of gambling engagement. Treatment approaches that have demonstrated efficacy for both PG and cannabis-use disorders (e.g., cognitive behavioral therapy) might be considered in this regard, although the extent to which these therapies are efficacious in adolescents warrants direct examination (Dennis et al., 2004; Ladouceur, Boisvert & Dumont, 1994).

### Limitations

The study has several important limitations. First, the cross-sectional design precludes causal determinations. For example, it cannot be discerned whether marijuana use leads to ARPG, ARPG leads to marijuana use, or additional factors (i.e. impulsivity) might link the two variables. Second, the sample is not nationally representative, so gener-alizability may be limited. Third, the survey-based methodology relies on self-report and is prone to responder bias leading to potential inaccuracies and under- or over-reporting of certain behaviors, especially illegal behaviors. Additionally, non-response may have contributed to biases and may limit generalizability. Fourth, several study measures, including ones assessing psychopathology, use non-diagnostic and dichotomous measurements. Using more precise measures with defined psychometric properties would be valuable in further understanding the relationships between substance use, ARPG and other measures. Additionally, problem-gambling severity was determined based upon self-reported criteria for PG whereas diagnostic interviews by a clinician represent the ‘gold standard’.

## CONCLUSIONS

Most adolescents have participated in past-year gambling, and gambling behaviors co-occur with substance use. The associations between marijuana use, problem-gambling severity and health/functional measures are consistent with previous research demonstrating heterogeneity of gambling behaviors and their correlates, and highlight the need for further studies of adolescent gambling and co-occurring substance-use behaviors.
